# Supplementary material for: MetaRibo-Seq measures translation in microbiomes
Source: Nat Commun. 2020 Jun 29;11:3268. doi: 10.1038/s41467-020-17081-z (PMC7324362; doi:10.1038/s41467-020-17081-z)
Supplement: Supplementary file 10 — Supplementary Data 7 [file 41467_2020_17081_MOESM10_ESM.zip › File2/Confidence_High_Taxonomy/181460_out.krona.html]

Javascript must be enabled to view this page.

members
magnitude
magnitudeUnassigned
count
unassigned
taxon
rank

181460\_out

2

2759
superkingdom
2

2
kingdom
4751

2
451864
subkingdom

2
phylum
4890

2
subphylum
147538

1
class
147550

222544
subclass
1

5139
order

SRS019685\_contig\_number\_20949
1

class
147545
1

1
subclass
451871

1
33183
order

1
34384
family

1
genus
34392

1

SRS015190\_contig\_number\_9296
species
63405
